# Forty percent of hospitalizations after severe sepsis are potentially preventable

**DOI:** 10.1186/cc14038

**Published:** 2014-12-03

**Authors:** HC Prescott, KM Langa, TJ Iwashyna

**Affiliations:** 1 University of Michigan, Department of Medicine and Institute of Healthcare Policy and Innovation, Ann Arbor, MI, USA; 2 VA Center for Clinical Management and Research, Ann Arbor, MI, USA; 3 Institute of Social Research, Ann Arbor, MI, USA

## Introduction

Patients are frequently rehospitalized in the 90 days after severe sepsis. The rate of readmission exceeds patients' baseline rate of hospitalization, and also exceeds the rate after matched nonsepsis hospitalizations [1]. We sought to determine the most common readmission diagnoses after severe sepsis, the extent to which readmissions may be preventable, and whether the pattern of readmission diagnoses differs from that of nonsepsis hospitalizations.

## Methods

We studied participants in the US Health and Retirement Study with linked Medicare claims (1998 to 2010) [2]. Using validated methods [3,4], we identified severe sepsis and nonsepsis hospitalizations, then measured 90-day readmissions in each cohort. Using Healthcare Cost & Utilization Project's Clinical Classification Software [5], we determined the 10 most common readmission diagnoses after severe sepsis. We measured rates of 'potentially preventable' readmissions using published definitions [6]. We compared rates of all-cause, potentially preventable, and cause-specific hospitalizations between survivors of severe sepsis and nonsepsis hospitalizations using chi-squared tests.

## Results

We identified 3,703 severe sepsis and 44,840 nonsepsis hospitalizations, of which 3,036 (82.0%) and 43,539 (93.1%) survived to discharge, respectively. In the next 90 days, 43.6% of severe sepsis survivors were rehospitalized, compared to 34.8% of nonsepsis survivors, *P *< 0.001. The top readmission diagnoses following severe sepsis (Table 1) included several recognized potentially preventable diagnoses: heart failure, pneumonia, exacerbation of chronic obstructive pulmonary disease (COPD), and urinary infection. Also common were readmissions for sepsis, acute renal failure, and aspiration pneumonitis, diagnoses that could plausibly be prevented or treated early to prevent hospitalization. Patterns of readmission differed in severe sepsis survivors; rates of readmission for sepsis, renal failure, respiratory failure, device complication, and aspiration pneumonitis were higher and accounted for a greater proportion of the total readmissions. Potentially preventable hospitalizations - infection (sepsis, pneumonia, urinary tract, and skin or soft tissue), heart failure, COPD exacerbation, acute renal failure, and aspiration pneumonitis - accounted for 40.5% of all readmissions after severe sepsis (compared to 25.8% following nonsepsis admission, *P *< 0.001), and 19.6% of severe sepsis survivors experienced a readmission for one of these diagnoses (compared to 9.5% following a nonsepsis admission, *P *< 0.001).

**Table 1:** Top ten hospitalization diagnoses in the 90 days following severe sepsis.

Rank	Diagnosis category	Proportion of all 90-day admissions (%)	Survivors with 90-day admission (%)
1	Congestive heart failure, nonhypertensive	10.4	5.7*
2	Septicemia	9.5*	6.5*
3	Pneumonia	5.4	3.5*
4	Rehabilitation care	5.1	3.2
5	Acute and unspecified renal failure	4.6*	3.2*
6	Respiratory failure	4.1*	2.5*
7	Complication of device, implant, or graft	3.5*	2.3*
8	COPD and bronchiectasis	3.1	1.8
9	Urinary tract infection	3.1	1.8*
10	Aspiration pneumonitis	2.8*	1.8*

*Value greater than that of nonsepsis survivors, *P *≤ 0.001 for each comparison.

## Conclusion

Forty percent of hospitalizations after severe sepsis occur for diagnoses that may be preventable. A few disease categories account for a relatively large proportion of the hospitalizations after severe sepsis, suggesting the feasibility of tailoring postdischarge interventions to patient's personalized risk for these common postsepsis diagnoses.
